# Climate Change and Children: Health Risks of Abatement Inaction, Health Gains from Action

**DOI:** 10.3390/children1020099

**Published:** 2014-08-14

**Authors:** Anthony J McMichael

**Affiliations:** The Australian National University, Canberra, ACT 0200, Australia; E-Mail: tony.mcmichael@anu.edu.au; Tel.: +1-2-6281-3326

**Keywords:** climate change, health, children

## Abstract

As human-driven climate change advances, many adults fret about the losses of livelihoods, houses and farms that may result. Children fret about their parents’ worries and about information they hear, but do not really understand about the world’s climate and perhaps about their own futures. In chronically worried or anxious children, blood cortisol levels rise and adverse changes accrue in various organ systems that prefigure adult-life diseases. Meanwhile, for many millions of children in poor countries who hear little news and live with day-to-day fatalism, climate change threatens the fundamentals of life—food sufficiency, safe drinking water and physical security—and heightens the risks of diarrhoeal disease, malaria and other climate-sensitive infections. Poor and disadvantaged populations, and especially their children, will bear the brunt of climate-related trauma, disease and premature death over the next few decades and, less directly, from social disruption, impoverishment and displacement. The recent droughts in Somalia as the Indian Ocean warmed and monsoonal rains failed, on top of chronic civil war, forced hundreds of thousands of Somali families into north-eastern Kenya’s vast Dadaab refugee camps, where, for children, shortages of food, water, hygiene and schooling has endangered physical, emotional and mental health. Children warrant special concern, both as children *per se* and as the coming generation likely to face ever more extreme climate conditions later this century. As children, they face diverse risks, from violent weather, proliferating aeroallergens, heat extremes and mobilised microbes, through to reduced recreational facilities, chronic anxieties about the future and health hazards of displacement and local resource conflict. Many will come to regard their parents’ generation and complacency as culpable.

## 1. Introduction

Human-driven climate change is now well and truly with us. It will have wide-ranging and mostly damaging or disruptive effects on the natural and built environments and on many of the human interactions with them. While the international community must hope and work for a stabilised global climate and for secure local living environments, there nevertheless remains the probability that, within three generations, the average global surface temperature will have risen by 3–4 degrees Celsius [1,2]. That magnitude and rapidity of average surface warming would have no precedent in the 200,000-year experience of *Homo sapiens.* We cannot realistically imagine what that world would either look or be like, nor how social, economic and political conditions might have changed in ways that would have many consequences for child wellbeing, health and, in some regions, physical survival.

In the foreseeable future children around the world will be at risk of many and various adverse health outcomes. In particular, these include: physical trauma from extreme weather events; mental and emotional health disorders, including those associated with the aftermath of weather disasters and with social disruptions, losses of livelihoods and displacement; under-nutrition and stunting; diarrhoeal, parasitic, vector-borne and other infectious diseases; and allergic respiratory disorders [3,4]. Social and emotional development will also be jeopardised in settings where climate change impacts degrade recreational amenities, grassed sports-grounds and easy patterns of social contact.

Among these various threats to health, children in lower-income countries, especially in tropical areas, will suffer the most from climate change due to further amplification of health risks by persistent poverty, crowded living, lack of access to clean water, poor sanitation and inadequate healthcare systems [5]. That, at least, is likely to be so for next decade or two [5]. However, as warming and shifts in rainfall advance and periods of extreme weather become more frequent, adverse health impacts will increasingly affect populations everywhere.

The main types of health risks from climate change, to children, are summarised in Box 1.

There are two important moral dimensions to concerns over the many likely adverse consequences for children of growing up in a climatically changing world. First, adults bear the age-old responsibility for the safety, security and healthy development of their children. Reinforcing that moral and instinctive obligation, we now also know that many of the physical, physiological and mental scars of adverse childhood experience will lead to lifelong health deficits.

Second, the current adult generation also has a prospective moral and perhaps self-interested responsibility to not impose unusual and perhaps extreme risk on future, more distant generations of children. Via our actions, short termism and social-cultural inertia, we are putting successive future generations of children at risk of being born into a world that has been degraded, destabilised, biologically impoverished and which is likely to have become un-inhabitable in many regions [6,7].

Consider the plight of the hundreds of young Somali children in the Dadaab refugee camps in northern Kenya, having fled with families in the wake of the confluence of brutal sectarian civil war and a prolonged drought that peaked in 2011. In light of the regional climatological research, the drought and its consequent under-nutrition and starvation are now widely attributed to ongoing global climate change. The warming of the western Indian Ocean sea surface has caused a shift in regional easterly wind patterns, including the annual monsoon rains in the Horn of Africa [8]. While hundreds of thousands of people have died, in conflict or because of the (conflict-exacerbated) drought and famine, an estimated 300,000 have fled and now live, or subsist, as refugees in Kenya and Ethiopia. The Dadaab refugee camps are now filled to four times their planned capacity, and half of the Somali refugees are children. Their livelihoods, education, full social development and future life options have been on hold for much of the past decade. While even the richest one-fifth of teenagers in Somalia has received, on average, less than six years of schooling, the poorest fifth, many of whom are now refugees, has received less than one year.

Box 1 **Main types of health risks to children from climate change (not exhaustive).**

Direct risks (primary):

- Extreme weather events: trauma, post-event infections, and mental/emotional impacts
- Extremes of heat: dehydration, exhaustion, heat stroke
- Fears, anxieties about future; social disengagement in later childhood
- Air pollutants (allergens, ozone, *etc.*) and respiratory disorders, including allergies

Less direct (secondary):

- Local and regional food shortages: stunting (physical, intellectual), starvation, increased infectious disease susceptibility
- Increased exposure to various climate-sensitive infectious diseases
  Diarrhoeal diseases (bacterial proliferation with warming)
  Cholera (flooding, crowding, with fecal contamination)
  Vector-borne infections: e.g., malaria (*falciparum* malaria causes many child deaths)

Diffuse health consequences (tertiary), often several steps removed from the initial climatic-environmental impact:

- Diverse health consequences of physical displacement (often into camps or slums) and resource-related conflicts
- Impaired recreational and sporting facilities and opportunities (too hot, surfaces too hard, lack of recreational ice and snow for high-latitude populations, *etc.*)
- Living in a biologically impoverished world (biodiversity and ecosystem losses): restricted emotional and aesthetic development and enjoyment

The numbers of displaced, sometimes orphaned, children will increase as climate change (along with a constellation of other mounting environmental and demographic pressures and stressors) itself increases. Estimates vary greatly, but many forecasts for mid-century and beyond, given current global emissions and warming trends, are now in the hundreds of millions [9,10,11]. The health risks for children of displaced families, forcibly relocated or desperately seeking refuge, are many and well documented [12,13]. Beyond the widespread risks to children of diarrhoeal and respiratory infections, of exposures to mosquito-borne infections, of food shortage and under-nutrition, with stunting of physical and intellectual development and blunting of immune system development and functioning, there is also a wide penumbra of mental and emotional health disorders.

Children fret about their parents’ worries and will often hear reference to the world’s increasing environmental problems and losses, although they usually cannot fully understand their import. They may worry also about their own futures. This type of chronic worry or anxiety typically causes blood cortisol levels to rise, initiating adverse change in various organ systems and prefiguring various adult-life cardiovascular and metabolic diseases. This includes an apparent influence of chronic or repeated emotional stress upon the maturation and subsequent functioning of the immune system [14]. This, in turn, may then increase susceptibility to contracting or suffering severely from various infectious diseases [15] and perhaps becoming more prone to respiratory allergies in response to pollens and spores (some sources of which will thrive under warmer and wetter conditions).

Elsewhere, for the many millions of children in poor countries who hear little news and live with day-to-day fatalism, climate change threatens the fundamentals of their existence—food sufficiency, safe drinking water and physical security. It also heightens risks of diarrhoeal disease, malaria and other climate-sensitive infectious diseases. Poor populations, and especially their children, will bear the brunt of climate-related illness, disease, trauma and premature death over the next few decades, including from climate-related social disruption, impoverishment and displacement [3].

### 1.1. Intrinsically High Health Risks in Children

As living organisms children have a greater sensitivity than do adults to many environmental exposures because of the needs of their developing and immature organ systems and the vulnerability of their immature cognitive and emotional regulation systems [15,16]. They have a high dependence on parents and other caregivers, whose levels of preparedness and response capacity may now be compromised by various social and economic aspects of climate change. Further, most of the modelling of the health impacts of future climate change under a range of plausible greenhouse emissions scenarios does not specifically include children. Indeed, and especially in lower-income countries, there are few large broad-spectrum datasets that measure child wellbeing and health in sufficient depth to monitor the possible detectable impacts of climate change [17].

The topic of climate change and child health faces a further challenge in the public discussion and policy-making realms since many find it difficult to understand the process and its consequences and, therefore, how and at what level to respond to it. Perhaps this explains the sparse reference, in diverse governmental and community child-focused policies, to the child health and development risks from climate change and its environmental consequences. There is also the perennial problem of short electoral time-horizons and our predilection for meeting near-term needs while taking little action on behalf of the future. As climate change proceeds, it will increasingly affect the oncoming generations; hence its main health consequences are not yet visible; and, for some, therefore not important or even credible.

It has been ironic that a primary focus of the UN’s Millennium Development Goals (2000–2015) has been on child wellbeing and health—reducing child illness, infectious diseases and premature death—and yet has made no substantive linking of those goals to Goal 7 (seeking better environmental management and water quality). The MDGs were framed in essentially itemised fashion, as a way of reducing existing social and health problems and inequities. Yet, the challenge that now looms, in a rapidly changing and uncertain world, is to head off some or all of the future risks to child health and its disparities within and among populations. This must be made an integral part of the currently incubating and more forward-looking UN Sustainable Development Goals, for 2016–2030.

### 1.2. Children as Transmitters of Knowledge and Understanding

There is another important aspect, distinct from viewing children as passive high-risk “targets” of climate change. They will soon become the adults of the 2030s and beyond, in societies striving to restore the world’s climate and the overall Earth system to normal composition and functioning. Part of the task, troglodyte opposition notwithstanding, is to infuse child education with a general, attractive and ecologically-based conceptualisation of the wonders and workings of the natural world, its fundamental underpinning of human wellbeing, health and survival and the fascinating interdependence of the natural biophysical processes that we and all life depend upon. Of course, the language, explanations and examples must be accessible and engaging. If done well, then not only will today’s generation of children understand the world very much better than did their parental predecessors, but from childhood onwards, they can help disseminate into the community a more enlightened understanding of the environmental necessities for, and the environmental consequences of, family life and daily living.

## 2. Mitigation: Child Health Co-Benefits

There is extensive ongoing research into the ‘collateral’ near-term co-benefits to health that accrue in national and regional populations whose governments take mitigation actions to abate greenhouse emissions and to increase the uptake (biosequestration) of atmospheric carbon dioxide [18]. However, there has been little particular focus on the co-benefits to child health. One important exception is the detailed research and evaluation in relation to the health, economic and climatic benefits of replacing traditional cook-stoves and their high-polluting low-grade fuels, prevalent in small unventilated housing in millions of poor rural and old suburban dwellings in South Asia, China and much of Central and South America. The resultant extreme levels of indoor air pollution cause an estimated 1.5 to two million deaths in women and children every year and contribute substantial amounts of greenhouse-potent carbon dioxide, black carbon and methane to the lower atmosphere [5]. That research has now led to major national initiatives in several countries to replace the old dirty technology with modern, fuel-efficient, clean technology.

Around most of the world there is the need and opportunity to phase out coal-fired power plants and internal combustion engines in car-congested cities. This will reduce the respiratory hazards to all exposed people, but particularly in young children with still-developing lungs.

A particularly important example of a health co-benefit for children is the increase in physical mobility and activity that reduced reliance on private cars would bring; a reliance that, for the moment, is rising rapidly in developing countries, such as China and India, along with surging rates of injury and deaths on the roads. By mid-century, car use in today’s high-income countries is predicted to be around 50 percent higher than now and essentially inelastic to changes in fuel prices and urban planning. In the emerging market countries, in contrast, car use is likely to rise around five-fold, although in those countries, the outcome will be much influenced by oil prices, land use, public transport and road building [19].

The provision of alternative modern public transport would contribute greatly to curtailing or averting the onset of serious problems of body overweight and frank obesity in childhood, now an epidemic problem in populations nearly everywhere and one which, with its accompanying metabolic disruptions, lays the foundations of various adult-life disease processes. Indeed, that particular health co-benefit would be further reinforced by changes in food production systems, moderate limits on red (ruminant) meat consumption and changes in the composition of social diets, all of which would relieve damaging pressures on local farming environments and pastures, reduce greenhouse gas emissions and lower the risks to human health from an excessive consumption of overly-processed and animal-based foods.

## 3. Conclusions

Faced with the gradually mounting risks to health from climate change, children warrant our special concern—both as children *per se* and as the coming generation likely to be exposed to increasingly extreme climate conditions later this century. Children face diverse risks that range from violent weather, heat extremes, proliferating aeroallergens and mobilised microbes through to reduced recreational facilities, chronic anxieties about the future and the health hazards of displacement and local resource conflict.

Yet, enlightened and rapid international mitigation action will reduce many of the local and regional pre-existing background risks to child health: obesity; metabolic dysfunction; respiratory disorders; and negative emotional states. That action itself will be motivated by an education system that infuses learning across the spectrum of curricular topics with ecological understanding and insights and helps create a future generation better informed and better able to reshape cultures and technologies within the sustainable limits of Earth’s biocapacity. Without such action, many will come to regard their parents’ generation and its complacency or ineptness as culpable.
